# Physical Activity Advice for Prevention and Rehabilitation of Low Back Pain- Same or Different? A Study on Device-Measured Physical Activity and Register-Based Sickness Absence

**DOI:** 10.1007/s10926-021-10005-8

**Published:** 2021-10-09

**Authors:** Nidhi Gupta, Charlotte Lund Rasmussen, Jan Hartvigsen, Ole Steen Mortensen, Els Clays, Ute Bültmann, Andreas Holtermann

**Affiliations:** 1 National Research Centre for the Working Environment, Lerso Parkalle 105, 2100 Copenhagen, Denmark; 2 Department of Sports Science and Clinical Biomechanics, University of Southern Denmark, Odense, Denmark; 3 Chiropractic Knowledge Hub, Odense, Denmark; 4 Department of Occupational- and Social Medicine, Holbæk Hospital, Copenhagen University Hospital, Holbæk, Denmark; 5 Department of Public Health and Primary Care, Ghent University, Ghent, Belgium; 6 Department of Health Sciences, Community and Occupational Medicine, University Medical Center Groningen, University of Groningen, Groningen, The Netherlands

**Keywords:** Compositional data analysis, Accelerometry, Blue-collar workers

## Abstract

*Purpose* We lack knowledge on whether the advice of “being physically active” should be the same for prevention and rehabilitation of low back pain (LBP). Sickness absence is a key outcome for LBP prevention and rehabilitation. We investigated the associations between physical activity and long-term sickness absence (LTSA) among employees with and without LBP. *Methods* Between 2011 and 2013, 925 Danish employees wore a Actigraph GTX3 accelerometer for 1–5 workdays to measure physical activity and reported LBP in past 7 days. Employees were followed for 4 years to determine their first register-based LTSA event (≥ 6 consecutive weeks). *Results* Among employees with LBP, increasing moderate-to-vigorous-intensity physical activity at work by 20 min and decreasing the remaining behaviors at work (ie., sitting, standing and light-intensity activity) by 20 min was associated with 38% (95% CI 17%; 63%) higher LTSA risk. Increasing light-intensity activity at work by 20 min and decreasing 20 min from the remaining behaviors was associated with 18% (95% CI 4%; 30%) lower risk. During leisure, increasing moderate-to-vigorous-intensity activity by 20 min or standing by 40 min was associated with 26% (95% CI 3%; 43%) lower and 37% (95% CI 0%; 87%) higher risk, respectively. Among employees without LBP, we found no such associations. *Conclusions* The physical activity advice ought to be different for LBP prevention and rehabilitation to reduce LTSA risk, and specified by domain and activity intensity. At work, employees with LBP should be advised to spend time on light-intensity physical activity and limit their time on moderate-to-vigorous-intensity physical activity. During leisure, employees should spend time on moderate-to-vigorous-intensity physical activity.

**Supplementary Information:**

The online version contains supplementary material available at 10.1007/s10926-021-10005-8.

## Introduction

Low back pain (LBP) is the leading global cause of work disability, imposing a large burden on employees, workplaces and societies [1]. A key driver of the economic burden of LBP is sickness absence. In the EU, LBP is responsible for almost 50% of all sickness absence (more than  three consecutive days) and the societal burden of LBP in Europe amounted to 441 billion EUR in 2016 [2, 3]. The burden of LBP is shown to be similar in the remaining part of the world [4]. Therefore, evidence-based knowledge on prevention and rehabilitation of LBP is urgently needed [5].

Advice to “be physically active (as tolerated)” plays a key role in the prevention and rehabilitation of LBP [6, 7]. However, there is uncertainty on the validity of this advice [8] and the evidence is not clear on whether the advice should be the same for both prevention and rehabilitation of LBP when aiming to prevent sickness absence.

One reason for the uncertainty of the advice could be that the advice does not distinguish between the domains (e.g., work or leisure) in which the physical activity is performed. We recently found, based on accelerometry, that physical activity at work was harmfully associated with long-term sickness absence (LTSA) [9–11], while physical activity during leisure (domestic, transport and spare time) was beneficially associated with LTSA [9]. Another reason for the uncertainty could be that the advice does not specify the intensity of physical activity. Previous studies have shown that higher intensity physical activity, compared to activities of lower intensity, seems to reduce musculoskeletal pain risk [12, 13] and thus sickness absence. However, evidence on how the intensity of physical activity influences sickness absence risk among employees with and without LBP is unclear.

Previous studies have often focused on only a single domain and/or on a specific intensity of physical activity during a day, such as only physical activity during leisure [14] or focusing only on moderate-to-vigorous physical activity (MVPA) [15]. However, it is likely that all intensities and domains of physical behaviors (i.e., physical activity of various intensities, sedentary behavior and sleep) throughout 24 h are important for LBP and sickness absence. In other words, it may be that not a single behavior (e.g., MVPA) or a single domain (e.g., work) is important for LBP and sickness absence, but how daily time is spent across the whole spectrum of domains and physical behaviors—such as sitting, standing, light physical activity (LIPA), MVPA, at work and during leisure, as well as sleep—taken together. Because a day is constrained to 24 h, we cannot increase time spent on one of these physical behaviors without decreasing time in at least one other behavior. Standard analytical techniques are not designed to address such co-dependent/constrained data. To analyze such data, a specialized analytical approach, compositional data analysis (CoDA), has been developed [16, 17]. CoDA addresses the co-dependency by transforming relative information between behaviors into log ratios (e.g., sedentary vs. non-sedentary), resulting in unconstrained data.

The aim of this study was to investigate the association between domain-specific physical behaviors and risk of LTSA among employees with and without LBP using the CoDA approach.

## Methods

This study included participants from the Physical wOrk DEmands and Prospective register-based Sickness Absence study (PODESA) cohort [9, 18] that was formed by merging two cohorts, NOMAD and DPhacto [19, 20]. More details on the background and harmonization of these two cohorts can be found elsewhere [18].

The DPhacto and NOMAD cohorts were approved by the Ethics Committee for the Capital Region of Denmark (file numbers H-2-2012-011, H-2-2011-047) [18]. Employees gave written consent for their participation and the use of the data for research studies.

A convenience sample was recruited from 22 workplaces engaged in construction, cleaning, garbage collection, manufacturing, assembling, transport, mobile plant operation and the health service sector.

This was a prospective study with 4-year follow-up from baseline. Baseline measurements consisted of accelerometry and questionnaires on demographics, work-related factors, and health. From baseline (2011–2013), each employee was followed-up for 4 years in the Danish “Register-based Evaluation of Marginalization’ (DREAM) [21] to determine their first event of LTSA.

### Accelerometry to Measure Physical Activity at Work and During Leisure Time

Employees wore a thigh-based GT3X+ accelerometer (ActiGraph, Florida) for 24 h consecutively for up to five workdays [20, 22]. Employees were asked to simultaneously fill-in a short paper-based diary reporting their time starting and ending work, going to and getting out of bed to sleep, and non-wear periods each day. The accelerometer data was processed using a MATLAB program Acti4 [23, 24] that accurately detects various postures and movements [23]. Using Acti4, time spent sedentary, standing, moving (standing with small movements that is not classified as walking), slow walking (< 100 steps/min), fast walking (≥ 100 steps/min), running, stair climbing, and cycling at work and leisure was determined. For the analysis, time spent moving and slow walking was combined to determine LIPA, while time spent fast walking, running and stair climbing was combined to determine MVPA. Time spent cycling was also added to MVPA during leisure. The following time periods were determined using information from the self-reported diary: time in bed (which was further visually checked for verification in Acti4); work period—hours spent at the participant’s primary occupation; and a leisure period defined as non-work periods (i.e., transport, secondary occupations, domestic, and spare time) excluding time in bed.

All non-working days and non-wear periods (their criteria detailed elsewhere, 20) were removed from the analyses. Employees who had at least 1 day with valid information on work, leisure and time in bed period were included in the analyses (criteria for a valid work, leisure and time in bed period are described in these studies, 19, 25–27].

For the analyses, work and leisure time spent sedentary, standing and on LIPA and MVPA and time in bed was averaged across all valid days 25, 28).

### Low Back Pain

Via an interview-based questionnaire at baseline, we collected information on whether employees had experienced LBP in the past 7 days with yes/no responses using a modified single item from the Nordic Musculoskeletal questionnaire [29, 30].

### Prospective Register-Based Long-Term Sickness Absence

Information on the first LTSA event during the 4-year follow-up was retrieved from the DREAM register. The DREAM register contains information on granted subsidized sickness absence/week for employees in Denmark [31]. In Denmark, the state pays sickness absence compensation to the employer after 30 continuous sick days. Therefore, DREAM contains information on sickness absence periods for ≥ 5 consecutive weeks. LTSA was defined as the occurrence of the first event (if any) that lasted for ≥ 6 consecutive weeks during the 4-year follow-up from baseline [32]. LTSA constitutes a major part of the substantial cost of sickness absence on workplaces and society, and is strong indicator of work disability and early retirement [33].

### Potential Confounders

The potential confounders were chosen a priori based on previous research on the association between physical behaviors and sickness absence [34, 35]. Age was determined using the person-unique civil registration number of employees. BMI was determined objectively by trained personnel. Smoking was determined using a single item with four responses which was then reduced to to smokers and non-smokers [9]. Duration of occupational lifting/carrying was determined using a single item with 6 responses ranging from ‘almost all the time’ to ‘never’ [28]. Information on employees’ vocational training and type of work were used as indicators of socioeconomic status (SES) [36, 37] and were summarized in three categories: white-collar, blue-collar-skilled, and blue-collar-unskilled [9]. Information on the pre-event of LTSA within 12 months was obtained from the DREAM register. We collected information on events of angina pectoris via interview-based questionnaire. Information on ‘influence at work’ was collected via two items from the Copenhagen Psychosocial Questionnaire [38] on a scale of 0 to 100% where 0 meant no influence at work.

### Statistical Analyses

The analyses were performed separately for those employees with and without LBP, following the CoDA approach [16, 39].

#### Main Analyses

Firstly, a 4-part composition of work time (sedentary, standing, LIPA and MVPA) and 5-part composition of leisure (sedentary, standing, LIPA, MVPA, and time in bed) were expressed as isometric log-ratios (*ilrs*) resulting in 3 and 4 *ilrs*, respectively (*ilrs* calculations in supplementary file A) [25]. Secondly, Cox proportional hazards regression models were fitted to the *ilrs* as exposures (in both domains) and the onset of LTSA as the outcome [39]. The model was adjusted for age, sex, BMI, smoking, occupational lifting/carrying.

In the Cox models, employees contributed data from baseline until the first event of LTSA occurred or until the end of a 4-year follow-up in case of no event. Forty-seven employees dropped out during the follow-up due to being emigrated, died, entered early retirement, entered ordinary retirement, or became pregnant (i.e. if going on maternity leave 8 months following baseline). These employees were censored (i.e., removed from the analysis) and contributed with the risk time in the analyses until the time of their drop out.

The assumptions of proportional hazards were met when tested by visual inspection and using the Grambsch-Therneau test [40]. The association between work and leisure compositions, respectively, and the risk of LTSA was assessed based on the statistical significance of Type-II likelihood-ratio tests. All results were considered significant if *p* ≤ 0.05.

As the Cox effect sizes expressed as *ilr* estimates (as shown in Table 2) are difficult to interpret, we used compositional isotemporal substitution models [28] to interpret the effect sizes. Specifically, this method enables predictions of difference in risks corresponding to changes in a given exposure compared to its reference; in our case, the composition of work time and leisure time were the exposures. Firstly, a reference composition was determined (i.e. the compositional mean of work time spent on physical behaviors). Secondly, based on the reference composition, new ‘theoretical’ work and leisure time compositions of physical behaviors were created by using the ‘one-to-remaining’ reallocation method [9, 28, 41]*.* Using this method, we incrementally increased/decreased the time spent in each physical behavior (sedentary, standing, LIPA and MVPA and time in bed) by decreasing/increasing time spent in the remaining behaviors equally according to their proportions, keeping the total time at work and leisure constant. For example, based on this method, 20 min of MVPA at work were reallocated to 7 min of standing, 4 min of LIPA, and 9 min of sedentary behavior at work while keeping the total work time constant. The new theoretical compositions can be seen in supplementary file B. These new theoretical compositions were then transformed into *ilrs* as explained above. Thirdly, the Cox parameter estimates were used to predict the hazard ratios and their 95% confidence intervals corresponding to these new theoretical compositions compared to their reference composition using the procedure explained in previous studies [9, 39]. Finally, the predicted hazard ratios (HRs) were plotted for employees with and without LBP. The corresponding 95% CI of the predicted HRs are presented in supplementary file B.

Similar compositional isotemporal substitution models were performed using the ‘one-to-one’ reallocation method where we created new theoretical compositions by incrementally increasing/decreasing the time spent on one behavior by decreasing/increasing the time in only one other behavior within the domain, keeping the total domain-specific time constant. We performed these specific reallocations to further understand the significant results based on the ‘one-to-remaining’ reallocation method.

#### Sensitivity Analyses

We performed the following sensitivity analyses to evaluate the robustness of the results obtained from the main analyses:

1. due to missing information on SES (n = 118), we performed two analyses with adjustment and no adjustment for SES among remaining 807 employees.
2. similarly, due to a technical error-related missing information on influence at work (n = 207), we performed two separate analyses adjusting and not adjusting for influence at work among remaining 718 employees.
3. due to missing information on angina pectoris (n = 173), we performed analyses including and excluding those that experienced angina pectoris (n = 13) among the remaining 752 employees.
4. we performed a separate analysis by excluding those employees with a pre-event of LTSA (n = 52).

## Results

### Sample and Descriptives

**Fig. 1:**
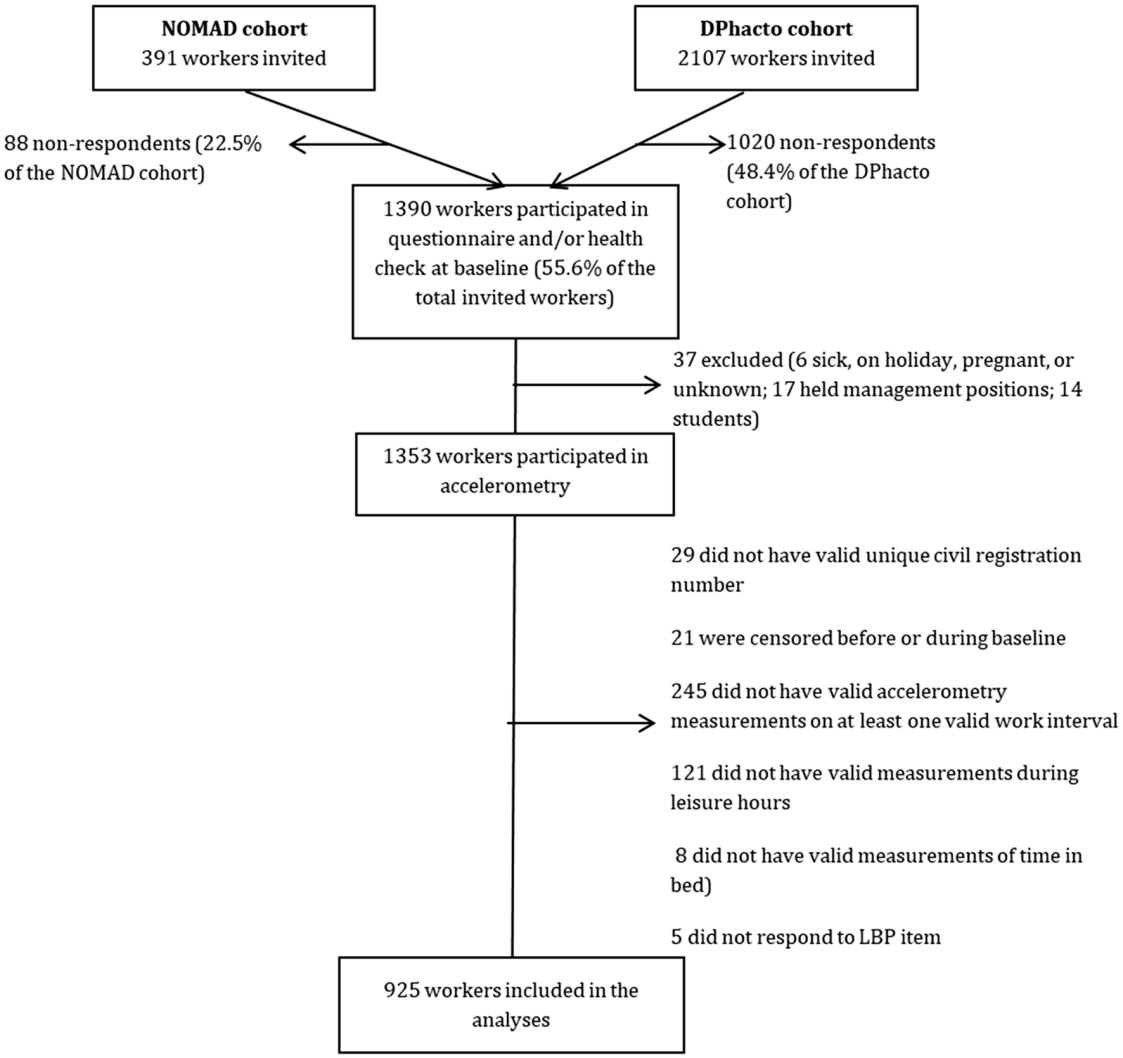
Flow of the participants

Of the 2,498 eligible participants, 925 (37%) filled-in the questionnaires, had valid accelerometry measurements, and provided their unique civil registration number to get information on LTSA from the DREAM register. A detailed flowchart can be found in Fig. 1.

Table 1 shows the baseline characteristics of the employees with LBP (n = 406) and without LBP (n = 519). Compared to employees without LBP (Table 1), employees with LBP reported longer occupational carry/lift duration, had lower influence at work and had slightly more blue-collar workers. Among employees with LBP, almost 23% had an event of LTSA, compared to 19% of the employees without LBP. No differences were observed for physical behaviors at work or during leisure between employees with and without LBP (Table 1).

**Table 1:** Baseline characteristics of the total participants (n = 925) and specifically for those with and without LBP at baseline

Variables	With LBP (n = 406)			Without LBP (n = 519)			Total (n = 925)		
	N	%^±^	Mean (SD)	N	%^±^	Mean (SD)	N	%^±^	Mean (SD)
Age (years)	406		44.8 (9.9)	519		45.0 (9.5)	925		44.9 (9.7)
Females	184	45		233	45		417	45	
BMI (kg/m^2^)	397		27.2 (4.8)	514		27.0 (4.8)	911		27.1 (4.8)
Smokers	122	30		152	30		274	30	
Occupational lifting/carrying duration (1–6)^≠^	405		3.7 (1.5)	519		4.0 (1.5)	924		3.9 (1.5)
Influence at work (0–100%)**	315		55.6 (27.5)	403		62.9(27.1)	718		60.6 (27.4)
White-collar	49	14		101	22		150	19	
Blue-collar skilled	147	42		173	38		320	40	
Blue-collar unskilled	158	45		179	40		337	42	
Job sector									
Cleaning	79	20		87	17		166	18	
Manufacturing	239	59		327	63		566	61	
Transport	24	6		38	7		62	7	
Health service	7	2		6	1		13	1	
Assemblers	8	2		15	3		23	3	
Construction	22	5		16	3		38	4	
Garbage collectors	14	3		9	2		23	3	
Mobile plant operators	8	2		2	0		10	1	
Others*	5	1		19	4		24	3	
Pre-event of LTSA 12 month prior baseline	23	6		29	6		52	6	
LTSA event in 4-year follow-up	93	23		97	19		190	21	
Angina pectoris (yes)	7	2		6	1		13	2	
Compositional means of time-use on physical behaviors (mins)									
Work	406	100	446	519	100	456	925	100	452
Sedentary	406	37	166	519	40	184	925	39	176
Standing	406	31	138	519	30	137	925	30	138
LIPA	406	18	79	519	15	71	925	16	74
MVPA	406	14	63	519	14	64	925	14	64
Leisure	406	100	899	519	100	885	925	100	892
Sedentary	406	35	316	519	35	307	925	35	312
Standing	406	9	82	519	8	74	925	9	77
LIPA	406	5	43	519	5	40	925	5	41
MVPA	406	4	32	519	4	33	925	4	33
Time in bed	406	47	426	519	49	432	925	48	429

*LBP* low back pain, *SES* socioeconomic status, *LTSA* long-term sickness absence, *LIPA* light physical activity, *MVPA* moderate-to-vigorous physical activity

^≠^1 almost all the time, 6 never; *general office clerks and other elementary workers: ** 0% meant no influence at work,  ^±^ for work and leisure physical behaviors, % represents the proportion of total measured work and leisure time

### Main Analyses

The work and leisure time compositions of physical behaviors were significantly associated with LTSA risk among employees with LBP (work, *p* = 0.001; leisure, *p* = 0.02) but not among those without LBP (work, *p* = 0.72; leisure, *p* = 0.85). Results of the estimates obtained from the Cox proportional hazards models are presented in Table 2.

**Table 2:** The Cox model-estimates associated with isometric log-ratios (ilrs) expressing the work (3 ilrs) and leisure (4 ilrs) time compositions of physical behaviors. Cox proportional hazards regression models were performed to investigate the association between work and leisure time composition of physical behaviors and risk of long-term sickness absence

Variable	With LBP (n = 406)			Without LBP (n = 519)		
	HR	95% CI	p	HR	95% CI	p
*Work*						
Ilr_1_[ln(MVPA:sedentary, stand,LIPA)]	3.12	1.73–5.62	< 0.001	0.72	0.38–1.37	0.31
Ilr_2_[ln(LIPA:stand, sedentary)]	0.63	0.37–1.09	0.10	1.15	0.68–1.93	0.61
Ilr_3_[ln(stand:sedentary)]	1.07	0.72–1.59	0.75	0.90	0.61–1.31	0.57
*Leisure*						
Ilr_1_[ln(MVPA:sedentary, stand,LIPA, time in bed)]	0.52	0.29–0.93	0.03	0.77	0.43–1.39	0.39
Ilr_2_[ln(sedentary:stand, LIPA, time in bed)]	1.31	0.64–2.68	0.47	1.11	0.52–2.38	0.78
Ilr_3_[ln(stand:LIPA, time in bed)]	2.17	0.85–5.52	0.11	0.78	0.32–1.86	0.57
lr_4_[ln(LIPA: time in bed)]	0.78	0.37–1.65	0.51	1.52	0.67–3.47	0.32

These estimates are from the Cox model where all ilrs from both work and leisure were included in the same model. Only estimates for *ilr*_*1*_ are interpretable as it contains information for the whole composition. *ilr*_*1*_ (MVPA) in the table represents the log ratio between MVPA as the numerator and the geometric mean of the remaining behaviors as the denominator. The estimates should be interpreted as one unit difference in LTSA risk corresponding to one unit change in each *ilr* adjusted for other ilrs and confounders

*LIPA* light physical activity, *MVPA* moderate-to-vigorous physical activity, *ilr* isometric log ratio, *HR* hazard ratio, *p* level of statistical significance

**Fig. 2:**
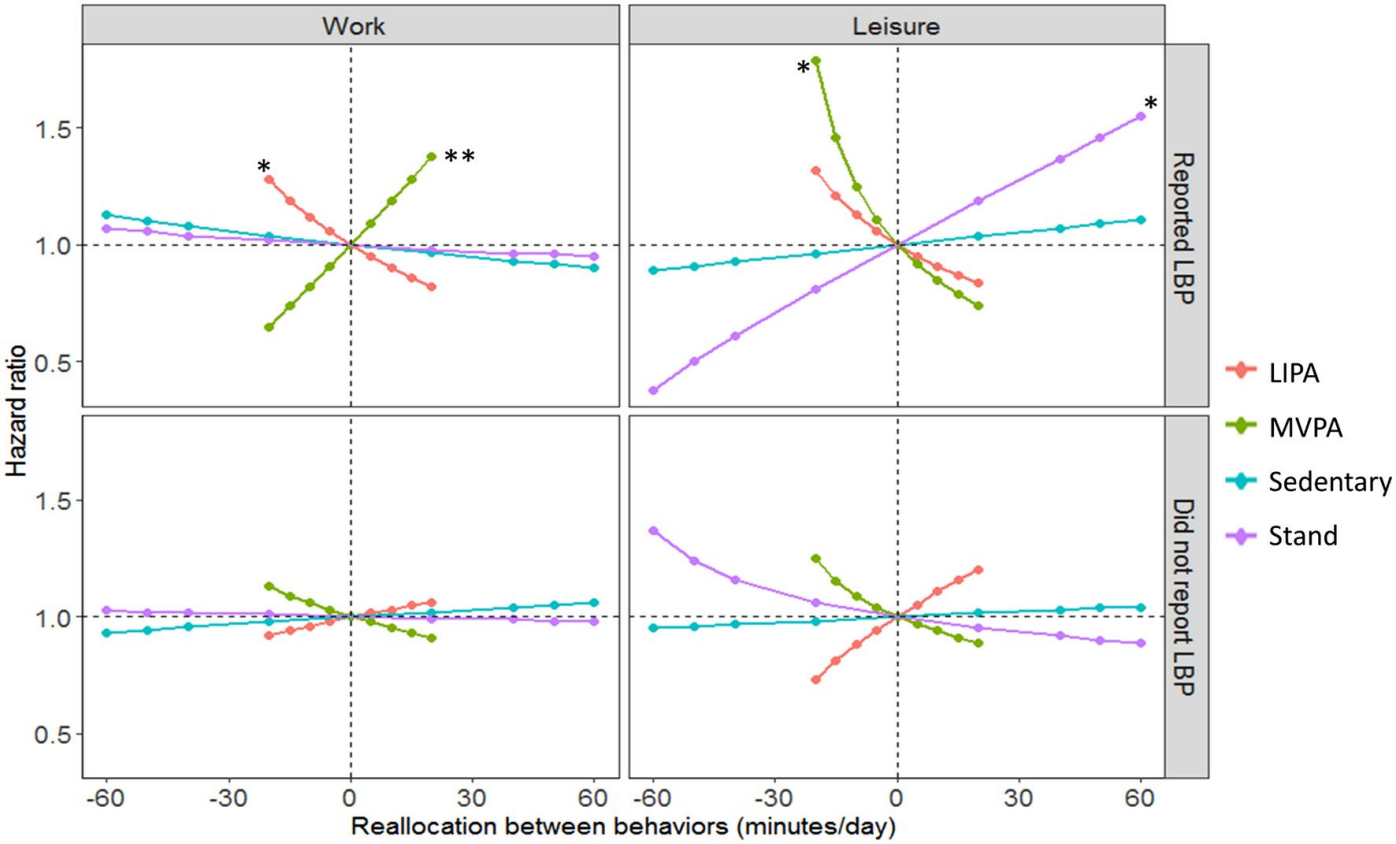
Results indicating how theoretical changes in the composition of work and leisure time spent on physical behaviors may influence LTSA risk among employees with (n = 406) and without LBP (n = 519). Results shown correspond to the *one-to-remaining reallocation* method: new theoretical compositions were created from the reference (mean) composition where the time in each physical behavior was theoretically increased/decreased by decreasing/increasing the time in the remaining behaviors, keeping the total domain time constant. Zero on the x-axis indicates reference composition (in minutes) of work (sedentary = 166, standing = 138, LIPA = 79, and MVPA = 63) and leisure (sedentary = 316, standing = 82, LIPA = 43, MVPA = 32 and, time in bed = 426). Number ‘1.0’ on y-axis represents unchanged LTSA risk corresponding to reference (mean) composition; hazard ratio indicates the difference between risk associated with the new composition and the reference composition; *indicates significant association at *p* ≤ 0.05 while ** indicates significant association at *p* ≤ 0.01. *LBP* low back pain, *LIPA* light intensity physical activity, *MVPA* moderate-to-vigorous physical activity

Among employees with LBP (Fig. 2), increasing work time spent on LIPA by decreasing time in the remaining behaviors at work (remaining behaviors are those getting replaced, in this case: sedentary, standing, and MVPA are getting replaced by LIPA at work) was associated with lower LTSA risk (eg., ↑20 min LIPA at work, and thus a corresponding ↓9 min sedentary, ↓8 min standing and ↓3 min MVPA, associated with ↓18% LTSA risk). In contrast, increasing MVPA at work by decreasing time in the remaining work behaviors was associated with higher risk (eg., ↑20 min MVPA at work associated with ↑38% risk).

During leisure (Fig. 2), increasing time spent standing, by decreasing time in the remaining behaviors was associated with an increased risk of LTSA (eg., ↑40 min standing was associated with a 37% ↑risk). Conversely, increasing time spent on MVPA, by decreasing time in remaining behaviors, was associated with a decreased LTSA risk (↑20 min leisure MVPA was associated with ↓26% risk).

#### One-to-One Reallocation

**Fig. 3:**
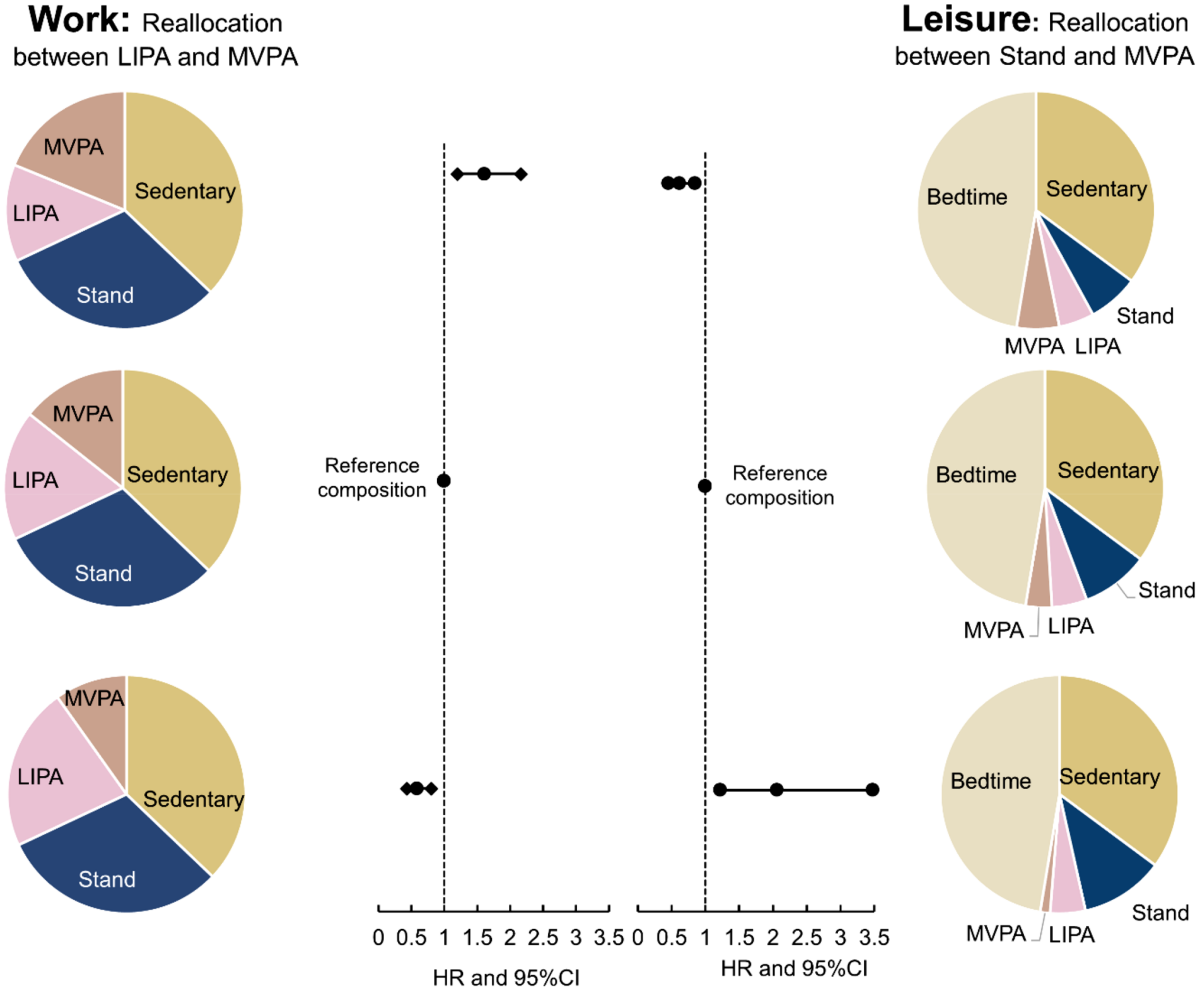
Theoratical results of the *one-to-one reallocation* method indicating the difference in LTSA risk corresponding to theoretically incrementally increasing/decreasing time between MVPA and LIPA at work and between MVPA and stand during leisure. The pie charts represent specific new theoretical work and leisure time compositions while the x axis represents the difference in LTSA risk (as HR) and its 95% confidence interval. *LIPA* light physical activity, *MVPA* moderate-to-vigorous physical activity

Figure 3 shows that at work, increasing 20 min MVPA by decreasing 20 min of LIPA was associated with 61% (HR 1.61, 95% CI 1.20–2.16) higher LTSA risk. During leisure, increasing 20 min MVPA by decreasing 20 min standing was associated with 38% (HR 0.62, 95% CI 0.45–0.85) lower LTSA risk.

#### Sensitivity Analyses

Overall, no large differences were observed between the main and the sensitivity analyses (to test confounding effect of SES, influence at work, angina pectoris and pre-event of LTSA) with respect to the magnitude and direction of the association between physical behaviors at work and leisure and LTSA risk (results not shown).

## Discussion

We investigated the association between domain-specific physical behaviors and risk of LTSA among employees with and without LBP. To the best of our knowledge, this is the first study to investigate this association using device-based measurements of 24-h time spent on work and leisure physical behaviors and using the CoDA approach. We observed that the composition of work and leisure-time spent on physical behaviors was associated with the risk of LTSA among employees with LBP, but not among employees without LBP. Specifically, we observed that among employees with LBP, at work, increasing relative MVPA time and decreasing relative LIPA time was associated with a higher risk of LTSA. Conversely during leisure, increasing relative MVPA time and decreasing relative standing time was associated with a lower risk of LTSA.

### Employees with LBP at Baseline

The observed harmful association between relative MVPA time at work and LTSA does not support the general rehabilitation advice to be physically active, as tolerated, when having LBP [6, 42]. A possible explanation for this result may lie in the characteristics of MVPA at work among blue-collar workers (i.e. 82% of the whole sample). These workers predominantly perform MVPA at work under job constraints which limit their possibility to tailor the duration, intensity, and variation of the MVPA according to their needs and preferences. Consequently, these workers may have insufficient recovery opportunity following the MVPA at work, which may lead to fatigue and aggravation of their LPB symptoms [43] increasing their risk for LTSA [44]. Our findings are in line with a recent review of device-based measures of physical behaviors and LBP that mainly included studies on blue-collar workers, observing harmful effects of occupational physical activity [45]. However, the studies involved in the review did not explore the associations with sickness absence, only with LBP. Our study thus extends the knowledge to prevent the risk of sickness absence among employees with LBP.

We also found that at work, increasing LIPA time by decreasing time on the remaining behaviors (standing, sedentary behavior, and MVPA) was associated with a lower LTSA risk. This result indicates that reducing all kinds of physical activities at work might not be the best rehabilitation advice to prevent LTSA, as physical activity of lower intensity (i.e. slow walking) at work seems to reduce the LTSA risk. Moreover, we observed the highest reduction of LTSA risk when increasing LIPA by decreasing MVPA at work (Fig. 3; ↑20 min LIPA and ↓20 min MVPA associated with a ↓41% LTSA risk). It is plausible that increasing LIPA by decreasing MVPA at work may provide the “just right” dose and type of physical activity for promoting musculoskeletal health and work participation among employees with LBP. Future longitudinal studies should focus on testing such hypotheses by including duration and type of all physical activity inside and outside of work and use statistical methods that are able to take into account all parameters, as used in this study [46].

During leisure, increasing MVPA time by decreasing time in the remaining leisure behaviors (i.e., sedentary, standing, LIPA, and time in bed) was associated with a reduced LTSA risk. This result supports the rehabilitation advice of being physically active to prevent LBP and sickness absence among employees with LBP [47]. Physical activity performed during leisure is often of an unconstrained nature [48]. Thus, it is during leisure that employees are more likely to be able to balance their MVPA time with periods of recovery according to their needs. This may provide the sufficient stimulus and recovery that is needed to obtain the musculoskeletal health benefits of physical activity [48].

We also found that during leisure, increasing relative time spent standing was associated with a higher LTSA risk. Although we did not have contextual information, these employees might be performing standing during leisure when engaged in household chores such as cooking or doing laundry. Because of spending more time standing, employees might be spending less time conducting beneficial physical activities, thus leading to higher LTSA risk. For example, in our study, we found that increasing standing by 20 min and decreasing MVPA by 20 min during leisure was associated with a 106% higher LTSA risk. More research using contextual information is required to understand how and why employees with LBP have an increased risk for LTSA from standing during leisure.

### Employees Without LBP at Baseline

Among employees without LBP, we found no statistically significant association between the leisure time composition of physical behaviors and LTSA risk. However, the association between relative time spent MVPA during leisure and LTSA seemed important (i.e., ↓20 min of MVPA by ↑time in remaining behaviors associated with ↑25% risk). Future studies using larger sample size are warranted to corroborate our findings. At work, we found no statistically significant associations between the time composition of physical behaviors and LTSA risk. Future studies are needed to confirm and understand the observed association between time spent on physical behaviors at work and LTSA risk among employees without LBP.

### Practical Implications

Our results indicate that, in order to prevent LTSA, the general prevention and rehabilitation advice on physical activity ought to be tailored to the state of LBP among employees. Additionally, the advice should take into account the intensity (e.g. MVPA, LIPA and standing) and domains (e.g. work or leisure) of physical behaviors. At work, employees with LBP should be given the opportunity to spend more time in LIPA and limit their MVPA time whereas during leisure, employees with LBP should be advised to spend more time in MVPA. Employees engaged in manual jobs (as in our study) generally perform more physical activity at work and are more fatigued compared to their peers in white-collar jobs [19]. Thus, it can be difficult to motivate them to follow physical activity advice. Future studies should address the structural and environmental interventions needed to successfully modify physical behaviors of employees in manual jobs to follow physical activity advice.

### Strengths and Limitations

The main strength of the study was the compositional data analysis (CoDA) approach that addresses the finite nature of physical behaviors data without violating statistical assumptions. Another strength of this study was the use of the national register to get information on LTSA events. National registers are shown to provide valid information on sickness absence when compared to company-based records [49] and self-reported sickness absence [50]. The use of thigh-based accelerometry, which enabled valid information on time spent on various physical behaviors within 24 h, was another strength of the study [23]. Moreover, the high adherence to accelerometry that provided, on average, 22.4 h of valid data each day for 1–5 working days increased representativeness of our data on physical behaviors.

A limitation of the study was the lack of objective information on other work and leisure physical behaviors that are difficult to measure using accelerometry, such as lifting at work. In line with this, the use of self-reported time in bed as a proxy of sleep time was also a limitation of the study. Inclusion of 37% of the total eligible sample in the statistical analyses was another limitation of the study. However, previous studies have indicated that there were no clear differences between participants and non-participants in the NOMAD and DPhacto cohorts used in this study [19, 51]. Another limitation is that we lacked information on LBP-specific sickness absence. We used the DREAM register that offers information on all-cause LTSA and not on cause-specific LTSA such as LBP-specific LTSA. Because of the high co-morbidity between LBP and other causes of LTSA, such as depression and anxiety [52], the validity of only LBP-specific LTSA can be questioned. Thus, we do not consider the absence of cause-specific LTSA to be a major limitation of our study. The lack of information on the interaction between employees with LBP and healthcare professionals was another limitation. It is likely that some of the employees with LBP may get an advice from the healthcare professionals to be physically active. This might have influenced the physical behaviors, particularly during leisure, of these workers. However, we did not see a clear difference in the physical behaviors during leisure between employees with and without LBP (Table 1).

## Conclusion

The occupational prevention and rehabilitation advice of physical activity ought to be different among employees with and without LBP to reduce risk of LTSA. Additionally, the advice should be specified on domain and intensity of the acitivities. However, intervention studies are required to test the effectiveness of such specified advices before being implemented.

## Supplementary Information

Below is the link to the electronic supplementary material.

Supplementary file1 (DOCX 32 kb)

## Data Availability

All data from NOMAD and DPhacto cohorts are now deposited in the National Archives collection of survey data (NOMAD: http://dda.dk/catalogue/28617?lang=da and DPhacto: http://dda.dk/catalogue/28618?lang=da) where data are available upon request.

## Code Availability

Codes are available upon request.
