# Supplementary material for: Physical Activity Advice for Prevention and Rehabilitation of Low Back Pain- Same or Different? A Study on Device-Measured Physical Activity and Register-Based Sickness Absence
Source: J Occup Rehabil. 2021 Oct 9;32(2):284–94. doi: 10.1007/s10926-021-10005-8 (PMC9232440; doi:10.1007/s10926-021-10005-8)
Supplement: Supplementary file 1 — Supplementary file1 (DOCX 32 kb) [file 10926_2021_10005_MOESM1_ESM.docx]

**SUPPLEMENTARY FILES**

**File A.** Calculation of ilrs used in the study.

To express the relative importance of sedentary behaviours during work, the ilr-coordinates of the 4-part work time-use composition were computed as

$$z_{1}^{*}=\sqrt{\frac{3}{4}}\ln\left( \frac{{MVPA}_{{work}_{i}}}{\sqrt[3]{{standing}_{{work}_{i}}*{sedentary}_{{work}_{i}}*{MVPA}_{{work}_{i}}}} \right)$$

$$z_{2}^{*}=\sqrt{\frac{2}{3}}\ln\left( \frac{{{LIPA}_{work}}_{i}}{{\sqrt[2]{{{standing}_{work}}_{i}*{sedentary}_{work}}}_{i}} \right)$$

$$z_{3}^{*}=\sqrt{\frac{1}{2}}\ln\left( \frac{{{standing}_{work}}_{i}}{{{sedentary}_{work}}_{i}} \right)$$

Giving rise to a work ilr-coordinate vector $ilr\left( \boldsymbol{Z} \right)=\left( \begin{matrix} z_{1}^{*} \\ z_{2}^{*} \\ z_{3}^{*} \end{matrix} \right)$ for each worker (*i*).

To express the relative importance of sedentary behaviours during leisure, the ilr-coordinates of the 5-part work time-use composition were computed as

$$y_{1}^{*}=\sqrt{\frac{4}{5}}\ln\left( \frac{{{MVPA}_{leis}}_{i}}{\sqrt[4]{{{standing}_{leis}}_{i}*{{LIPA}_{leis}}_{i}*{{sedentary}_{leis}}_{i}*{time in bed}_{i}}} \right)$$

$$y_{2}^{*}=\sqrt{\frac{3}{4}}\ln\left( \frac{{{sedentary}_{leis}}_{i}}{\sqrt[3]{{{LIPA}_{leis}}_{i}*{{standing}_{leis}}_{i}*{time in bed}_{i}}} \right)$$

$$y_{3}^{*}=\sqrt{\frac{2}{3}}\ln\left( \frac{{{standing}_{leis}}_{i}}{\sqrt[2]{{{LIPA}_{leis}}_{i}*{time in bed}_{i}}} \right)$$

$$y_{4}^{*}=\sqrt{\frac{1}{2}}\ln\left( \frac{{{LIPA}_{leis}}_{i}}{{time in bed}_{i}} \right)$$

Giving rise to a leisure time ilr-coordinate vector $ilr\left( \boldsymbol{Y} \right)=\left( \begin{matrix} y_{1}^{*} \\ y_{2}^{*} \\ y_{3}^{*} \\ y_{4}^{*} \end{matrix} \right)$ for each worker (*i*).

**File B**. 95% confidence intervals of the hazard ratios indicating the difference in LTSA risk corresponding to incrementally increasing/decreasing time between MVPA and LIPA at work and between MVPA and stand in leisure (results from one-to-remaining reallocations as shown in Figure 1).

| Reported LBP | | | | | | Did not report LBP | | | | | |
| --- | --- | --- | --- | --- | --- | --- | --- | --- | --- | --- | --- |
| lowCI | HR | HighCI | Reallocation | Behavior | domain | lowCI | HR | HighCI | Reallocation | Behavior | domain |
| 0.97 | 1.13 | 1.32 | -60 | Sedentary | Work | 0.81 | 0.93 | 1.07 | -60 | Sedentary | Work |
| 0.97 | 1.1 | 1.25 | -50 | Sedentary | Work | 0.85 | 0.94 | 1.06 | -50 | Sedentary | Work |
| 0.98 | 1.08 | 1.19 | -40 | Sedentary | Work | 0.88 | 0.96 | 1.04 | -40 | Sedentary | Work |
| 0.99 | 1.04 | 1.09 | -20 | Sedentary | Work | 0.94 | 0.98 | 1.02 | -20 | Sedentary | Work |
| 1 | 1 | 1 | 0 | Sedentary | Work | 1 | 1 | 1 | 0 | Sedentary | Work |
| 0.92 | 0.97 | 1.01 | 20 | Sedentary | Work | 0.98 | 1.02 | 1.06 | 20 | Sedentary | Work |
| 0.85 | 0.93 | 1.02 | 40 | Sedentary | Work | 0.96 | 1.04 | 1.13 | 40 | Sedentary | Work |
| 0.82 | 0.92 | 1.03 | 50 | Sedentary | Work | 0.95 | 1.05 | 1.17 | 50 | Sedentary | Work |
| 0.79 | 0.9 | 1.03 | 60 | Sedentary | Work | 0.94 | 1.06 | 1.2 | 60 | Sedentary | Work |
| 0.8 | 1.07 | 1.44 | -60 | Stand | Work | 0.76 | 1.03 | 1.39 | -60 | Stand | Work |
| 0.83 | 1.06 | 1.34 | -50 | Stand | Work | 0.8 | 1.02 | 1.3 | -50 | Stand | Work |
| 0.87 | 1.04 | 1.26 | -40 | Stand | Work | 0.85 | 1.02 | 1.22 | -40 | Stand | Work |
| 0.94 | 1.02 | 1.11 | -20 | Stand | Work | 0.92 | 1.01 | 1.1 | -20 | Stand | Work |
| 1 | 1 | 1 | 0 | Stand | Work | 1 | 1 | 1 | 0 | Stand | Work |
| 0.91 | 0.98 | 1.06 | 20 | Stand | Work | 0.92 | 0.99 | 1.08 | 20 | Stand | Work |
| 0.82 | 0.96 | 1.13 | 40 | Stand | Work | 0.84 | 0.99 | 1.15 | 40 | Stand | Work |
| 0.79 | 0.96 | 1.16 | 50 | Stand | Work | 0.81 | 0.98 | 1.19 | 50 | Stand | Work |
| 0.75 | 0.95 | 1.19 | 60 | Stand | Work | 0.78 | 0.98 | 1.23 | 60 | Stand | Work |
| 1.05 | 1.28 | 1.56 | -20 | LIPA | Work | 0.75 | 0.92 | 1.14 | -20 | LIPA | Work |
| 1.03 | 1.19 | 1.38 | -15 | LIPA | Work | 0.81 | 0.94 | 1.1 | -15 | LIPA | Work |
| 1.02 | 1.12 | 1.23 | -10 | LIPA | Work | 0.87 | 0.96 | 1.06 | -10 | LIPA | Work |
| 1.01 | 1.06 | 1.11 | -5 | LIPA | Work | 0.94 | 0.98 | 1.03 | -5 | LIPA | Work |
| 1 | 1 | 1 | 0 | LIPA | Work | 1 | 1 | 1 | 0 | LIPA | Work |
| 0.91 | 0.95 | 0.99 | 5 | LIPA | Work | 0.97 | 1.02 | 1.06 | 5 | LIPA | Work |
| 0.83 | 0.9 | 0.98 | 10 | LIPA | Work | 0.95 | 1.03 | 1.13 | 10 | LIPA | Work |
| 0.76 | 0.86 | 0.97 | 15 | LIPA | Work | 0.92 | 1.05 | 1.19 | 15 | LIPA | Work |
| 0.7 | 0.82 | 0.96 | 20 | LIPA | Work | 0.9 | 1.06 | 1.26 | 20 | LIPA | Work |
| 0.53 | 0.65 | 0.81 | -20 | MVPA | Work | 0.89 | 1.13 | 1.43 | -20 | MVPA | Work |
| 0.63 | 0.74 | 0.86 | -15 | MVPA | Work | 0.92 | 1.09 | 1.29 | -15 | MVPA | Work |
| 0.74 | 0.82 | 0.91 | -10 | MVPA | Work | 0.95 | 1.06 | 1.18 | -10 | MVPA | Work |
| 0.87 | 0.91 | 0.96 | -5 | MVPA | Work | 0.97 | 1.03 | 1.08 | -5 | MVPA | Work |
| 1 | 1 | 1 | 0 | MVPA | Work | 1 | 1 | 1 | 0 | MVPA | Work |
| 1.04 | 1.09 | 1.14 | 5 | MVPA | Work | 0.93 | 0.98 | 1.02 | 5 | MVPA | Work |
| 1.09 | 1.19 | 1.29 | 10 | MVPA | Work | 0.87 | 0.95 | 1.05 | 10 | MVPA | Work |
| 1.13 | 1.28 | 1.46 | 15 | MVPA | Work | 0.81 | 0.93 | 1.07 | 15 | MVPA | Work |
| 1.17 | 1.38 | 1.63 | 20 | MVPA | Work | 0.76 | 0.91 | 1.09 | 20 | MVPA | Work |
| 0.74 | 0.89 | 1.08 | -60 | Sedentary | Leisure | 0.78 | 0.95 | 1.17 | -60 | Sedentary | Leisure |
| 0.78 | 0.91 | 1.06 | -50 | Sedentary | Leisure | 0.81 | 0.96 | 1.14 | -50 | Sedentary | Leisure |
| 0.82 | 0.93 | 1.05 | -40 | Sedentary | Leisure | 0.85 | 0.97 | 1.11 | -40 | Sedentary | Leisure |
| 0.91 | 0.96 | 1.02 | -20 | Sedentary | Leisure | 0.92 | 0.98 | 1.05 | -20 | Sedentary | Leisure |
| 1 | 1 | 1 | 0 | Sedentary | Leisure | 1 | 1 | 1 | 0 | Sedentary | Leisure |
| 0.98 | 1.04 | 1.1 | 20 | Sedentary | Leisure | 0.95 | 1.02 | 1.08 | 20 | Sedentary | Leisure |
| 0.95 | 1.07 | 1.21 | 40 | Sedentary | Leisure | 0.91 | 1.03 | 1.17 | 40 | Sedentary | Leisure |
| 0.94 | 1.09 | 1.27 | 50 | Sedentary | Leisure | 0.89 | 1.04 | 1.21 | 50 | Sedentary | Leisure |
| 0.93 | 1.11 | 1.32 | 60 | Sedentary | Leisure | 0.87 | 1.04 | 1.26 | 60 | Sedentary | Leisure |
| 0.14 | 0.38 | 0.99 | -60 | Stand | Leisure | 0.43 | 1.37 | 4.34 | -60 | Stand | Leisure |
| 0.25 | 0.5 | 1 | -50 | Stand | Leisure | 0.57 | 1.24 | 2.72 | -50 | Stand | Leisure |
| 0.37 | 0.61 | 1 | -40 | Stand | Leisure | 0.67 | 1.16 | 2 | -40 | Stand | Leisure |
| 0.65 | 0.81 | 1 | -20 | Stand | Leisure | 0.85 | 1.06 | 1.33 | -20 | Stand | Leisure |
| 1 | 1 | 1 | 0 | Stand | Leisure | 1 | 1 | 1 | 0 | Stand | Leisure |
| 1 | 1.19 | 1.4 | 20 | Stand | Leisure | 0.8 | 0.95 | 1.13 | 20 | Stand | Leisure |
| 1 | 1.37 | 1.87 | 40 | Stand | Leisure | 0.67 | 0.92 | 1.26 | 40 | Stand | Leisure |
| 1 | 1.46 | 2.12 | 50 | Stand | Leisure | 0.61 | 0.9 | 1.32 | 50 | Stand | Leisure |
| 1 | 1.55 | 2.39 | 60 | Stand | Leisure | 0.57 | 0.89 | 1.38 | 60 | Stand | Leisure |
| 0.77 | 1.32 | 2.26 | -20 | LIPA | Leisure | 0.39 | 0.73 | 1.37 | -20 | LIPA | Leisure |
| 0.84 | 1.21 | 1.75 | -15 | LIPA | Leisure | 0.53 | 0.81 | 1.24 | -15 | LIPA | Leisure |
| 0.9 | 1.13 | 1.41 | -10 | LIPA | Leisure | 0.68 | 0.88 | 1.14 | -10 | LIPA | Leisure |
| 0.95 | 1.06 | 1.18 | -5 | LIPA | Leisure | 0.83 | 0.94 | 1.06 | -5 | LIPA | Leisure |
| 1 | 1 | 1 | 0 | LIPA | Leisure | 1 | 1 | 1 | 0 | LIPA | Leisure |
| 0.87 | 0.95 | 1.05 | 5 | LIPA | Leisure | 0.95 | 1.05 | 1.17 | 5 | LIPA | Leisure |
| 0.76 | 0.91 | 1.09 | 10 | LIPA | Leisure | 0.9 | 1.11 | 1.36 | 10 | LIPA | Leisure |
| 0.67 | 0.87 | 1.13 | 15 | LIPA | Leisure | 0.86 | 1.16 | 1.55 | 15 | LIPA | Leisure |
| 0.6 | 0.84 | 1.17 | 20 | LIPA | Leisure | 0.83 | 1.2 | 1.75 | 20 | LIPA | Leisure |
| 1.07 | 1.79 | 3 | -20 | MVPA | Leisure | 0.76 | 1.25 | 2.05 | -20 | MVPA | Leisure |
| 1.04 | 1.46 | 2.04 | -15 | MVPA | Leisure | 0.83 | 1.15 | 1.6 | -15 | MVPA | Leisure |
| 1.03 | 1.25 | 1.53 | -10 | MVPA | Leisure | 0.9 | 1.09 | 1.32 | -10 | MVPA | Leisure |
| 1.01 | 1.11 | 1.21 | -5 | MVPA | Leisure | 0.95 | 1.04 | 1.14 | -5 | MVPA | Leisure |
| 1 | 1 | 1 | 0 | MVPA | Leisure | 1 | 1 | 1 | 0 | MVPA | Leisure |
| 0.85 | 0.92 | 0.99 | 5 | MVPA | Leisure | 0.89 | 0.97 | 1.04 | 5 | MVPA | Leisure |
| 0.73 | 0.85 | 0.98 | 10 | MVPA | Leisure | 0.81 | 0.94 | 1.08 | 10 | MVPA | Leisure |
| 0.64 | 0.79 | 0.97 | 15 | MVPA | Leisure | 0.74 | 0.91 | 1.12 | 15 | MVPA | Leisure |
| 0.57 | 0.74 | 0.97 | 20 | MVPA | Leisure | 0.69 | 0.89 | 1.16 | 20 | MVPA | Leisure |
